# Exercise training does not mitigate high‐phosphate diet‐induced mitochondrial dysfunction in skeletal muscle in male mice

**DOI:** 10.14814/phy2.71139

**Published:** 2026-10-19

**Authors:** Hannah F. Sanders, Dominic W. Kolonay, Jade A. Blackwell, Jiaru Liu, Isabella R. Dobrski, Daniel A. Branch, Lucas A. Lefers, Benjamen H. Kern, Kedryn K. Baskin

**Affiliations:** ^1^ Department of Physiology and Cell Biology The Ohio State University Columbus Ohio USA; ^2^ Dorothy M. Davis Heart and Lung Research Institute The Ohio State University Wexner Medical Center Columbus Ohio USA

**Keywords:** exercise, high‐phosphate diet, mitochondrial respiration, skeletal muscle metabolism

## Abstract

Inorganic phosphate consumption in adults is two‐to‐three‐fold higher than the recommended daily allowance due to its widespread use as a preservative and flavor enhancer in processed foods. We previously reported that 12 weeks of high‐phosphate diet (HPD) reduced VO_2max_ and altered metabolic gene expression and mitochondrial dysfunction in mouse skeletal muscle. Here, we investigated whether exercise training reverses the negative consequences of HPD. Male mice were fed normal (NPD) or HPD for 5 weeks, then continued on NPD or HPD with 9 weeks of voluntary exercise training. Serum, urine, and fecal phosphate increased within 1 day of HPD‐feeding and remained elevated throughout the study. Trained NPD‐ and HPD‐fed mice performed similarly on forced exercise tests, but mitochondrial respiration was significantly decreased in skeletal muscle from trained HPD‐fed mice. RNA sequencing revealed distinct sets of differentially expressed genes when comparing sedentary versus exercise‐trained muscle of HPD‐fed mice relative to the same comparison in NPD‐fed mice. These findings indicate that voluntary exercise training preserves endurance but does not fully restore gene expression or mitochondrial function in muscle from HPD‐fed mice. Thus, HPD‐induced skeletal muscle dysregulation is not completely reversible with exercise, underscoring the importance of both diet and exercise for metabolic health.

## 1 INTRODUCTION

Inorganic phosphate (Pi) is widely used as a preservative and flavor enhancer in processed foods, resulting in U.S. adults consuming two‐to‐three times more Pi than the recommended daily allowance of 700 mg (Supplements NIoHOoD, n.d.; Calvo et al., 2014; U.S. Department of Agriculture ARS, 2024; Wolfson et al., 2025). Chronic high Pi intake is associated with reduced renal function and kidney damage, decreasing filtration capacity and increasing the risk of developing chronic kidney disease (CKD) (Rubio‐Aliaga & Krapf, 2022). Persistent excess dietary Pi induces vascular calcification in both healthy individuals and patients with CKD, which in turn contributes to the development of hypertension and cardiovascular disease (Calvo & Uribarri, 2013). Long‐term excess Pi consumption also decreases bone mass and bone density (Calvo & Uribarri, 2013; Ugrica et al., 2021; Vorland et al., 2017). Thus, sustained Pi overload disrupts multiple physiological systems, creating a metabolic environment vulnerable to dysfunction.

Chronic Pi overconsumption represents a significant dietary threat with far‐reaching consequences for metabolic health. In previous studies, we demonstrated that a short‐term 12‐week HPD impacts metabolically active skeletal muscle tissue, reduces exercise duration and maximal oxygen consumption (VO_2max_) in mice independent of cardiac dysfunction or other chronic HPD‐related pathologies. RNA sequencing of gastrocnemius plantaris (GP) skeletal muscle revealed carbohydrate metabolism‐related genes and fatty‐acid metabolism‐related genes were significantly increased and decreased, respectively. Isolated GP mitochondria from HPD‐fed mice displayed reduced oxygen consumption when provided with substrates succinate or palmitoyl carnitine (PC), indicating lower respiratory capacity (Peri‐Okonny et al., 2019). Together, these data indicate that short‐term HPD remodels skeletal muscle metabolism by dysregulating metabolic gene expression and inducing mitochondrial dysfunction, likely contributing to decreased exercise capacity.

Aerobic exercise training remodels systemic metabolism across multiple tissues (Ahn, Hevener, et al., 2025; Ashcroft et al., 2024; Montgomery et al., 2023; Murphy et al., 2020). Key features of white adipose tissue (WAT) remodeling include enhancing lipolysis, inducing beiging, and increasing adiponectin secretion, which collectively improves insulin sensitivity in peripheral tissues (Ahn, Zhang, et al., 2025; Garritson & Boudina, 2021; McKie & Wright, 2020; Nigro et al., 2023). In the liver, exercise increases fatty acid oxidation (FAO), which reduces hepatic triglyceride content, and hepatic insulin sensitivity increases, which improves glucose and gluconeogenesis regulation (Handy & Holloway, 2023; Hargreaves & Spriet, 2020; McGee & Hargreaves, 2020). Skeletal muscle becomes more insulin sensitive due to glycogen depletion and increased glucose transporter expression (Jensen et al., 2011; Leal et al., 2018). Exercise also elevates muscle lipolysis and shifts muscle fibers towards oxidative metabolism, stimulating mitochondrial biogenesis (Kelahmetoglu et al., 2020; Smith et al., 2023). The vast beneficial effects of aerobic exercise on human health are reflected in the prescription of “exercise is medicine” (Cartee et al., 2016; Sallis, 2009; Sawalla Guseh et al., 2022).

Here, we tested the hypothesis that exercise training can mitigate the negative effects of HPD on skeletal muscle metabolism. Male mice were first fed either a normal phosphate diet (NPD) or HPD for 5 weeks and then randomized into sedentary or voluntary exercise‐training groups while consuming the same diet for an additional 9 weeks. We assessed training by measuring the average daily distance run and evaluated exercise endurance using a forced treadmill test in both sedentary and trained groups. To determine how diet and exercise influenced skeletal muscle metabolism, we measured respiration of isolated GP mitochondria. Finally, we investigated molecular mechanisms underlying HPD‐ and exercise‐induced adaptations by analyzing differentially expressed genes (DEGs) and Reactome Pathways from RNA sequencing data.

## 2 MATERIALS AND METHODS

### 2.1 Mouse studies

All animal experiments were approved by and conducted under The Ohio State University Institutional Animal Care and Use Committee. Mice were housed in a pathogen‐free barrier facility with a 12‐h light/dark cycle at room temperature. To model excess dietary Pi intake in humans (U.S. Department of Agriculture ARS, 2024; Lee & Cho, 2015), we fed adult male mice a normal‐phosphate diet (NPD, 0.6% wt/wt Pi, *n* = 15) or high‐phosphate diet (HPD, 2.0% wt/wt Pi, *n* = 15) (Figure S1a) based on our prior study (Peri‐Okonny et al., 2019). 25‐week‐old C57BL6 male randomized mice were fed ad libitum either HPD (Inotiv, TD.150280) or a nutrient‐ and mineral‐matched normal phosphate diet (NPD) (Inotiv, TD.250301) for 14 weeks. Chow weights were recorded weekly to determine food intake. Throughout the study, measurements were collected in a blinded manner.

### 2.2 Body composition measurements

Total fat mass and lean body mass were measured using an EchoMRI™100H (EchoMRI LLC) body composition analyzer according to manufacturer instructions. Body weights were recorded weekly and were normalized to tibia length, collected and measured at the end of the study.

### 2.3 Voluntary wheel‐running exercise training

After 5 weeks on diet, randomized mice were subjected to voluntary exercise training using in‐cage mouse running wheels (Starr Life Sciences Corp.). Eight mice underwent exercise training for both NPD and HPD. Wheel revolutions were collected every 15 min using Starr Life Sciences VitalView Activity Software, and data is presented as the average kilometers run per day and per week. Trained mice had access to wheels until the end of the study (nine‐weeks of training total).

### 2.4 Treadmill exercise tests

After 14 weeks of diet, exercise duration was assessed in a blinded manner by a graded treadmill exercise test using an Exer 3/6 Treadmill (Columbus Instruments) after fasting for 2–5 h at the end of the dark cycle. This differs from the metabolic treadmill used in our previous study (Peri‐Okonny et al., 2019). Mice were acclimated to the treadmill two independent days before the exercise test. Acclimation consisted of running 10 m/min for 5 min and 20 m/min for another 5 min with no incline and with use of a shock grid fixed at 45 and 2 Hz. Exercise exhaustion tests were performed using the following protocol: 10 m/min for 5 min without incline, 20 m/min for 5 min without incline, 20 m/min for 5 min with a 10‐degree incline. The incline was kept at 10 degrees, and the treadmill speed increased 1 m/min every 5 min. Mice were run until exhaustion, defined as ten continuous seconds of shock grid contact with failure to continue running three consecutive times (Serrano et al., 2025).

### 2.5 Blood lactate measurements

Blood lactate concentration was measured at rest (an hour before testing) and immediately after treadmill exercise tests using the Lactate Plus lactate meter and strips (Nova Biomedical). Blood was obtained via tail vein puncture.

### 2.6 Inorganic phosphate (Pi) quantification

Serum, urine, and fecal Pi levels were analyzed using the Phosphate Assay Kit (BioChain Institute Inc., Z5030017) according to the manufacturer's instructions. Briefly, blood, urine, and feces were collected at the beginning of the light cycle. Blood was drawn via mandibular bleed and centrifuged at 1000 × *g* for 10 min at 4°C to obtain serum. Feces were placed in 1.5 mL Eppendorf tubes, dried in a non‐CO_2_ incubator for 24 h at 60°C, and dissolved in 0.6 M hydrochloric acid for another 24 h (Ugrica et al., 2021). Fecal and urine samples were diluted 1:1000 and serum samples were diluted 1:10 for Pi quantification using a quarter of the manufacturer recommended reagent and sample volumes, as optimized.

### 2.7 Mitochondria isolation and functional analysis

Gastrocnemius plantaris (GP) muscles and liver were used for isolated mitochondria function analysis. Use of isolated mitochondria facilitates controlled analysis of mitochondrial function outside of cellular metabolic influence (Heller et al., 2012; Rogers et al., 2011). After dissection, one GP muscle and the right lateral lobe of the liver were immediately placed in mitochondrial isolation buffer (MHSE) on ice. GP muscle was minced before being placed in MHSE buffer. MHSE is composed of 70 mM sucrose, 210 mM mannitol, 1 mM HEPES, 1 mM EGTA, and 0.5% fatty acid‐free bovine serum albumin (BSA; MP Biomedical, ICN15240150). Mitochondria were isolated using differential centrifugation, as previously described (Peri‐Okonny et al., 2019; Rogers et al., 2011). Briefly, tissues were disrupted in MHSE buffer using a size 23 KIMBLE® Potter‐Elvehjem tissue grinder and glass pestle with 4–6 strokes at 2500 rpm at 4°C. The tissue homogenates were then centrifuged at 800 × *g* for 10 min at 4°C. The resulting supernatants were filtered through a 40 μm Nylon cell strainer (Falcon) followed by centrifugation at 8000 × *g* for 10 min at 4°C. The pellets obtained after centrifugation were resuspended in MHSE buffer and centrifuged again at 8000 × *g* for 10 min at 4°C. The resulting pellets were resuspended in 1 mL of MSHE without BSA and centrifuged for a final time at 8000 × g for 5 min at 4°C. The final pellets were resuspended in 50 and 100 μL of MSHE without BSA for GP and liver, respectively. The Bradford assay was used to determine the quantity of mitochondrial protein present in all samples (Bradford, 1976) (Bio‐Rad, 5000205).

The Seahorse XFe96 Analyzer (XFe96 Seahorse Bioscience, Agilent) was used to measure oxygen consumption rates (OCR) to assess mitochondrial function of GP and liver mitochondria. Electron‐coupling (EC) and electron‐flow (EF) assays were performed in tandem (Rogers et al., 2011). The EC assay enables measurement of respiratory states 2 (basal), 3 (active respiration in response to ADP), State 4_O_ (leak, in response to oligomycin), and 3_U_ (maximal respiration in response to FCCP), while the EF assay assesses sequential respiration of electron transport chain (ETC) Complex I through Complex IV (CI‐CIV) in uncoupled mitochondria. EC assays were performed in two assay buffers, optimized for tissue source: KCl for GP and MAS for liver, supplemented with 0.2% (w/v) fatty acid‐free BSA (MP Biomedical), and 10 mM succinate (Sigma S2378) and 2 μM rotenone (Sigma R8875) were provided as substrates. KCl buffer is composed of 250 mM KCl, 4 mM KH_2_PO_4_, 4 mM MgCl_2_·6H_2_O, 40 mM HEPES, and 0.08 mM EGTA, adjusted to pH 7.2. MAS buffer is composed of 140 mM sucrose, 440 mM mannitol, 10 mM KH_2_PO_4_, 10 mM MgCl_2_·6H_2_O, 4 mM HEPES, and 2 mM EGTA, adjusted to pH 7.2. EF assays were performed in MAS buffer supplemented with 0.2% (w/v) fatty acid‐free BSA for both GP and liver. 10 mM pyruvate (sodium pyruvate, Sigma S8636), 2 mM malate (Sigma M6413), and 4 μM carbonyl cyanide 4‐(trifluoromethoxy)phenylhydrazone (FCCP, Sigma C2920) were provided as substrates. For fatty acid oxidation (FAO) assessment, 60 μM PC (Cayman 11095), 0.4 mM malate, and 4 μM FCCP were provided in EF assay buffer. Isolated mitochondria were quantified by Bradford assay. 6 and 12 μg of GP mitochondria for all experimental samples were plated for EC and EF assays, respectively. 7 μg of liver mitochondria were plated for both EC and EF assays. All conditions, including the amount of mitochondria, the assay buffer compositions, substrate and injection reagent concentrations, were optimized for GP and liver before performing assays on experimental samples to ensure isolated mitochondria from all tissues met the limit of detection of the instrument (Rogers et al., 2011). Mitochondria were diluted in respective assay buffers for EC and EF assays supplemented with BSA and 25 μL was plated per well in a Seahorse XFe96 cell culture microplate. The microplate was centrifuged at 2000 × g for 20 min at 4°C, and mitochondria presence was verified by bright field microscopy after centrifugation. EC or EF assay buffer supplemented with BSA were added to appropriate samples for a final well volume of 180 μL to perform the assays. OCR measurements were taken at baseline and throughout the assay using the following protocol: mix for 1 min, then measure for 3 min, three sequential times. EC assay reagents were injected in sequential order as follows: (1) adenosine diphosphate (ADP, Sigma A2754) at 2 and 4 mM for GP and liver, respectively, (2) 3 μM oligomycin (oligo, Sigma O4876), (3) FCCP at 4 and 12 μM for GP and liver, respectively, and (4) 4 μM antimycin A (AA, Sigma A8674) and 4 μM rotenone. EF assay reagents were injected in sequential order as follows: (1) 2 μM rotenone, (2) 100 μM succinate, (3) 4 μM AA, and (4) 150 μM N, N, N′, N′, tetramethyl‐*p*‐phenylenediamine (TMPD, Sigma T7394). OCR measurements are reported as picomoles of oxygen consumed per minute (pmol/min/μg protein).

### 2.8 RNA isolation, quantification, sequencing and analysis

Total RNA was extracted from GP muscle using Tri‐Reagent (Sigma T9424) according to the manufacturer's protocol, and RNA concentration was determined using NanoDrop One Spectrophotometer (ThermoFisher Scientific). GP samples from NPE and NPE groups (*n* = 4–5) containing 900 ng of total RNA with an RNA Integrity Number of at least 8 was submitted for RNA sequencing by Plasmidsaurus using the Illumina platform. Raw sequencing reads were obtained as fastq files and quality checks were performed with FastQC (v0.12.0) and trimmed using TrimGalore (v0.6.11). Trimmed reads were aligned using the default parameters of Spliced Transcripts Alignment to a Reference (STAR, v2.7.11b). Read counts were generated from sorted BAM files (SAMtools v1.23) using featureCounts default settings (Subread v2.1.1). Differential expression analysis was performed on sequencing from NPE and HPE samples and GSE120958 using DESeq2 (v1.50.2) and normalized for sequencing depth. The Wald test was used to determine statistical significance, and adjusted *p* values were generated using a Benjamini‐Hochberg false discovery rate. Genes surpassing thresholds of significance (adjusted *p* value *p*_adj_ <0.05) were exported and used for further analysis. Principal component analysis (PCA) was performed in R and heatmaps were generated from *z*‐scores of dysregulated genes using pheatmap (v1.0.13) using expression thresholds of |log_2_(fold change)| >1.5. Gene set enrichment analysis was performed using Enrichr (https://maayanlab.cloud/Enrichr/enrich) (Xie et al., 2021) to identify reactome pathways enriched between experimental groups using less stringent expression thresholds of |log_2_(fold change)| >0.5 to capture pathway enrichment. The overlapping differentially expressed genes (DEGs) between the four comparisons were visualized using the Venn diagram tool (https://bioinfogp.cnb.csic.es/tools/venny) (Oliveros & Venny, n.d.).

### 2.9 DNA extraction and quantification

DNA was isolated from GP and quantified as previously described (Peri‐Okonny et al., 2019). Briefly, tissue was homogenized in Tri‐Reagent (Sigma T9424), chloroform was added, and phase separation was performed by centrifugation. After removal of the RNA‐containing aqueous phase, back extraction buffer (1 M tris(hydroxymethyl)aminomethane, 4 M guanidine thiocyanate, 50 mM sodium citrate) was added to the interphase and organic phase. Mixed samples were incubated at room temperature and centrifuged at 3000 × g for 30 min at 4°C. The DNA was precipitated from the aqueous phase using isopropanol with polyacryl carrier (Molecular Research Center, Inc. PC 152). DNA was washed with 75% ethanol, dried, and resuspended in 8 mM NaOH with 10 mM HEPES and 1 mM EDTA. qPCR was performed in a CFX384 Real Time System (BioRad) with iTaq SYBR Green Universal Supermix (BioRad 1725120), 5 ng of DNA, and validated primers to detect Mitochondrial 16S rRNA (mtDNA) and Lipoprotein Lipase (Lpl), a readout of nuclear DNA (nDNA) (Peri‐Okonny et al., 2019; Quiros et al., 2017). DNA copy number was analyzed using the ΔΔCt method and data are reported as mtDNA/nDNA calculated by log_2_(2^−ΔΔCt^) (Peri‐Okonny et al., 2019; Quiros et al., 2017).

### 2.10 Western blot

Frozen isolated mitochondria were thawed on ice and boiled at 95°C in Laemmli buffer containing 2.5% 2‐mercaptoethanol for 5 min. 5 μg of total mitochondrial protein was loaded for all samples into pre‐cast 4%–20% Stain Free gradient gels (BioRad, 4561096). All Blue protein ladder (BioRad, 1610373) was used for molecular weight identification. Gels were run at 75 V for 15 min followed by 125 V for 50 min. Wet transfers were performed at 90 V for 90 min using methanol‐activated PVDF membranes (Milipore Immobilon, IPFL00010). Ponceau stain (Sigma‐Aldrich, P7170) was used to confirm equal protein loading. Membranes were blocked in 5% non‐fat dry milk in 1X PBS/0.1% Tween‐20 for an hour before incubation with total OXPHOS rodent antibody cocktail (1:500, Abcam, ab110413) overnight at 4°C. Membranes were washed in PBS‐T and incubated with HRP‐conjugated secondary mouse antibody (1:10,000, Jackson ImmunoResearch, 115‐0530003) for 1 h at room temperature. Imaging was performed using a ChemiDoc MP Imaging System (BioRad, 12003154) and BioRad Clarity and ClarityMax ECL substrate (BioRad, 1705060 and 1705062). Western blot quantification was performed as previously described (Kolonay et al., 2025).

### 2.11 Statistical analysis

Unpaired *t*‐tests were used to compare the following: (1) body composition, (2) serum, urine, and fecal Pi levels, (3) average kilometers run per day on wheels, and (4) OCR measurements in EC and EF assays. 2‐way ANOVA was performed on average kilometers ran per week in wheels and pre‐ and post‐run blood lactate measurements. Unpaired *t*‐tests and 2‐way ANOVA were performed with GraphPad Prism version 10.0 (GraphPad Software, Inc). Grubbs' outlier tests were performed, and outliers were removed accordingly. Spearmen's Rank Correlation was performed to explore the relationship between GP mitochondrial respiration and the average daily distance run in wheel cages. All data are presented as mean ± the standard deviation (SD).

## 3 RESULTS

### 3.1 Pi loading in mice occurs within the first 24 h of consuming a high phosphate diet

Previous studies have shown that HPD increases serum, urine, and fecal Pi, but have not assessed how quickly Pi loading occurs (Knopfel et al., 2017; Komuro et al., 2024; Oda et al., 2023; Tani et al., 2007). To determine when increased Pi levels can be detected, we collected serum, urine, and feces for 2 days after HPD introduction (Figure S1b). Serum Pi is significantly increased in HPD‐fed mice (8.1 ± 1.4 mg/dL) compared to NPD‐fed mice (5 ± 0.03 mg/dL) 1 day after diet initiation (Figure 1a). Serum Pi remains significantly increased 2 days post‐diet introduction (~5 mg/dL higher than NPD‐fed mice) (Figure 1a). Urine from HPD‐fed mice contained eight‐fold higher Pi levels than NPD‐fed mice on the first and second day after diet initiation (Figure 1b). Fecal Pi was three‐fold higher in HPD‐ compared to NPD‐fed mice on the first and second day after diet introduction (Figure 1c). Collectively, HPD induces effective Pi loading in mice within the first 24 h of consuming the diet.

**FIGURE 1:**
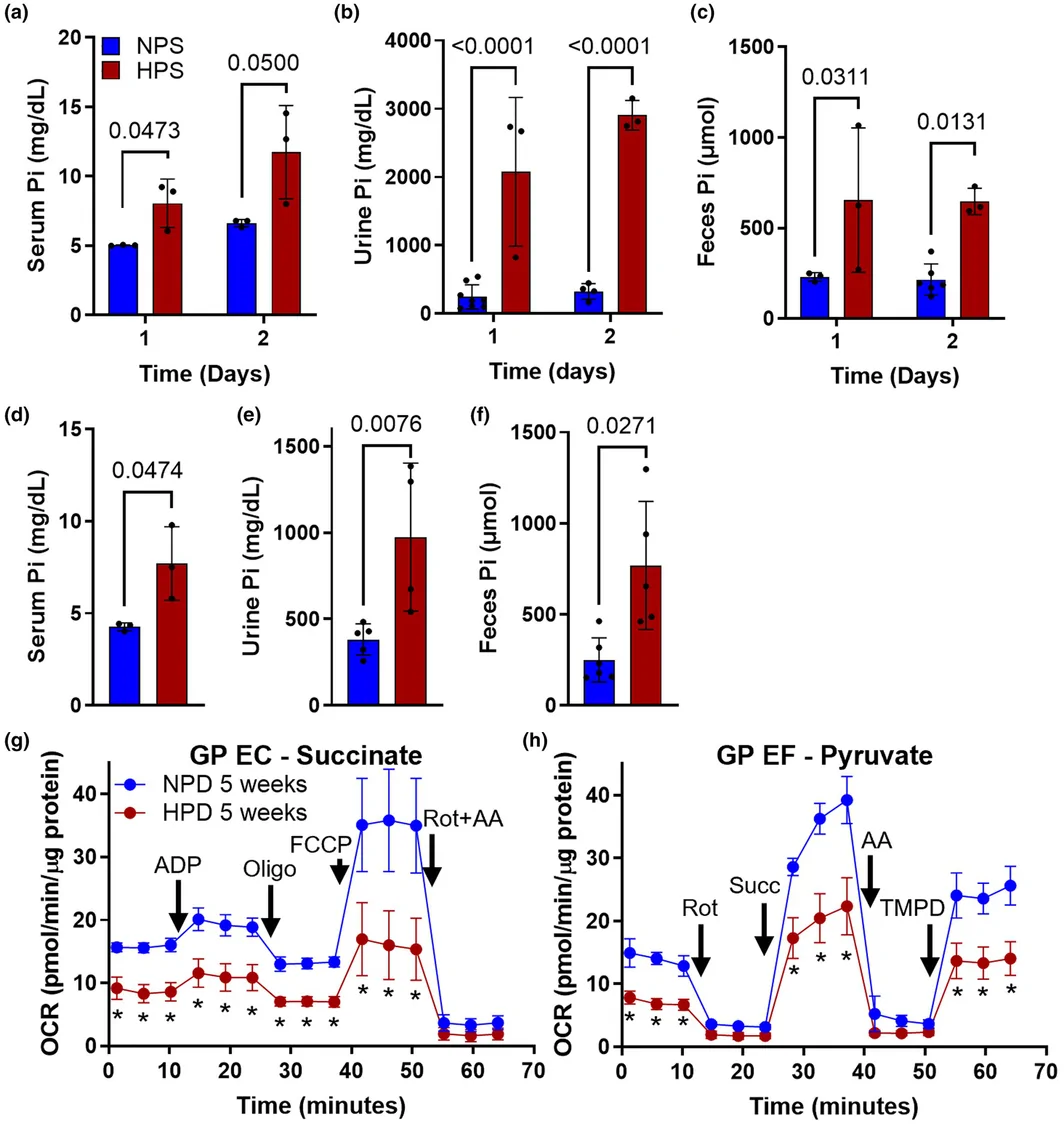
Phosphate loading occurs quickly after consumption of high‐phosphate diet (HPD), impairing muscle mitochondrial function after 5 weeks. (a) Serum, (b) urine, and (c) fecal inorganic phosphate (Pi) levels after initiation of HPD. (d) Serum, (e) urine, and (f) fecal Pi levels 5 weeks after initiation of HPD. (g) Coupled respiration of gastrocnemius plantaris (GP) isolated mitochondria provided with 10 mM succinate and 2 μM rotenone. (h) Uncoupled respiration of GP isolated mitochondria provided with 10 mM pyruvate, 2 mM malate, and 4 μM FCCP. *n* = 3–6 mice/day/condition. Data are mean ± SD. *p* determined by unpaired Student's *t*‐test.

### 3.2 Five weeks of high phosphate diet reduces skeletal muscle mitochondrial respiration

Serum Pi was significantly increased in mice fed HPD compared to NPD after 5 weeks on diet (Figure 1d) and phosphate clearance was maintained as urinary Pi and fecal Pi levels from HPD‐fed mice were significantly higher than NPD‐fed mice (Figure 1e,f). In earlier studies we observed that 12 weeks of HPD impairs skeletal muscle mitochondrial respiration (Peri‐Okonny et al., 2019). To determine whether a shorter HPD regimen impacts skeletal muscle mitochondrial function, we measured respiration in mitochondria isolated from gastrocnemius plantaris (GP) muscles after 5 weeks on diet. GP muscle mitochondria isolated from HPD‐fed mice have significantly decreased State 3 and 3_U_ respiration compared to GP mitochondria isolated from NPD‐fed mice when provided with succinate under coupled conditions (Figure 1g and Table S1). HPD GP muscle mitochondrial respiration of CII‐CIV and through CIV is significantly decreased in uncoupled conditions when given pyruvate as substrate (Figure 1h). Liver mitochondria isolated from HPD‐fed mice do not have significantly different respiration in coupled or uncoupled conditions compared to NPD‐fed mice (Figure S2 and Table S1), indicating liver dysregulation does not occur at 5‐weeks, similar to 12‐weeks in our previous study. Thus, 5 weeks of HPD is sufficient to induce mitochondrial dysfunction in GP muscle.

### 3.3 Exercise training initiation does not affect Pi loading

To determine if exercise can mitigate the negative consequences of HPD, we subjected mice to voluntary exercise training after 5 weeks of HPD and maintained them on HPD for an additional 9 weeks during training. We collected serum, urine, and feces 2 days after initiating voluntary wheel running to assess whether exercise alters serum, urine, and fecal Pi levels. After 2 days of voluntary exercise, mice fed HPD (HPE) had significantly increased serum Pi compared to mice fed NPD (NPE) (Figure 2a). HPE mice had significantly higher urinary and fecal Pi than NPE mice (Figure 2b,c). Importantly, 2 days of exercise training did not affect serum, urinary, or fecal Pi levels (Figure 1d–f and Figure 2a–c), demonstrating that acute exercise does not substantially affect Pi homeostasis in mice.

**FIGURE 2:**
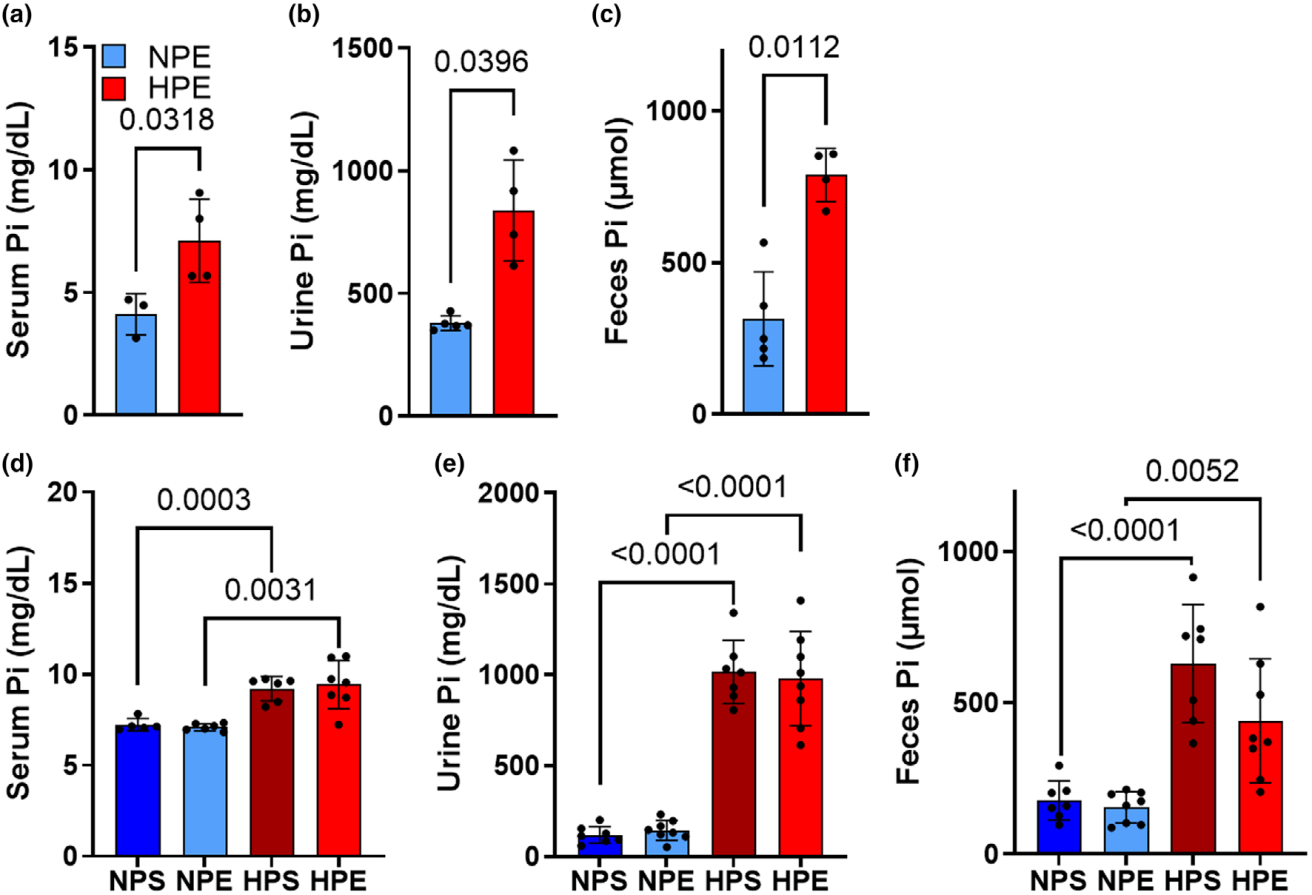
High‐phosphate diet (HPD) significantly increases inorganic phosphate levels in serum, urine, and feces. (a) Serum, (b) urine, and (c) fecal inorganic phosphate (Pi) levels 5 weeks after initiation of HPD and 2 days after initiation of voluntary wheel running. (d) Serum, (e) urine, and (f) fecal Pi after 14 weeks on diet. *n* = 3–8 mice/day/condition. Data are mean ± SD. *p* determined by unpaired *t*‐test.

Consistent with our previous findings, serum Pi remained significantly increased in HPD fed mice after 9 weeks of voluntary exercise training (HPE); however, there was no difference in serum Pi between the sedentary and exercise groups within each diet (Figure 2d). We also observed that after 14 weeks of HPD, mice have sustained ability to clear excess dietary phosphate, indicated by significantly increased urinary and fecal Pi (Figure 2e,f). Additionally, we did not observe differences in Pi clearance between sedentary or trained mice fed NPD or HPD (Figure 2e,f). Ultimately, exercise training does not affect serum, urine, or fecal Pi levels, and HPD provides effective dietary Pi loading for 14 weeks.

### 3.4 High phosphate diet does not alter body composition

We did not observe significant differences in weekly body weights between normal‐phosphate sedentary (NPS) and high‐phosphate sedentary (HPS), or NPE and HPE mice (Figure 3a). To determine if HPD altered body composition in trained mice, we measured total lean and fat mass after 14 weeks of diet. There was no significant difference in lean and fat mass between mice fed HPD and NPD in either sedentary or exercised groups (Figure 3b,c). Trained mice had significantly increased lean mass and decreased fat mass compared to sedentary mice, indicating effective exercise training regardless of diet (Figure 3b,c). GP muscle from trained mice had significantly increased mitochondrial DNA content, indicative of mitochondrial biogenesis that occurs with exercise training (Figure S3a). There were no significant differences in food consumption between the four groups (Figure 3d). Thus, Pi intake does not affect body composition in sedentary or exercised NPD or HPD mice, and HPD does not alter body composition in trained mice.

**FIGURE 3:**
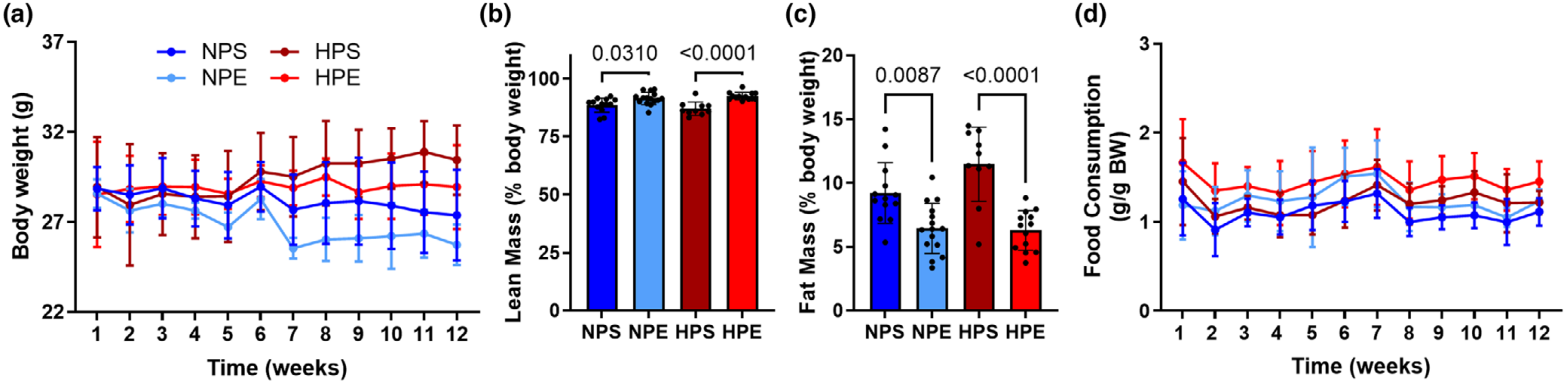
High‐phosphate diet does not affect body weight, body composition, or food consumption in sedentary or exercised mice. (a) Body weight normalized to tibia length. (b) Lean mass and (c) fat mass at the end of the study. (d) Food consumption normalized to body weight. *n* = 7–12 mice/condition. Data are mean ± SD. *p* determined by one‐way ANOVA.

### 3.5 High phosphate diet does not affect exercise duration in trained mice

To determine if exercise intervention effectively reduces or reverses HPD‐induced skeletal muscle metabolic dysregulation, mice were provided with access to voluntary wheels 5 weeks after diet initiation. The average daily distance ran each week did not differ between groups (Figure 4a). Overall, NPE and HPE mice ran similar distances on average per day throughout the entire study (Figure 4b), suggesting that HPD does not affect voluntary exercise training.

**FIGURE 4:**
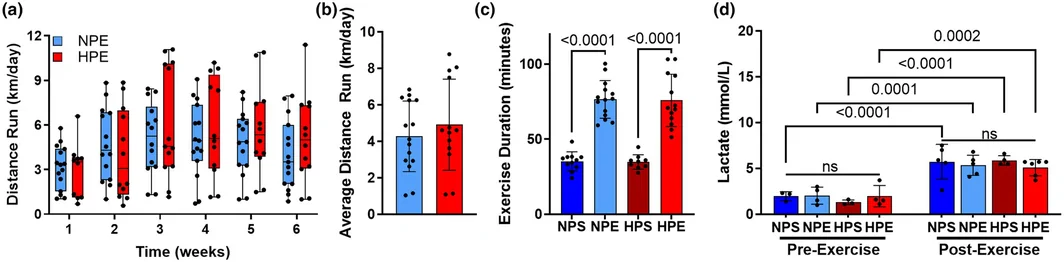
High‐phosphate diet does not affect voluntary exercise training or forced exercise duration. (a) Average daily distance run each week in voluntary wheel‐running cages. (b) Average kilometers run per day throughout the exercise study. (c) Exercise duration determined by forced exercise treadmill tests. (d) Blood lactate measurements before and after treadmill exercise tests. *n* = 7–14 mice/condition. Data are mean ± SD. *p* determined by one‐way ANOVA for exercise duration and 2‐way ANOVA for blood lactate measurements.

We next determined whether HPD affects exercise duration in trained NPD‐ and HPD‐fed mice. Body weight and composition were similar across groups, so we did not expect differences in exercise duration to be attributable to weight. As anticipated, trained mice ran longer than their sedentary counterparts, and blood lactate levels significantly increased after testing in all mice, regardless of diet (Figure 4c,d). Pre‐ and post‐exercise lactate measurements were not significantly different across all groups. HPD did not impair exercise duration in trained mice, as NPE and HPE mice ran for a similar amount of time during exercise treadmill tests (Figure 4c).

### 3.6 Voluntary exercise training does not rescue HPD‐induced muscle mitochondrial dysfunction

We next assessed isolated GP and liver mitochondrial function of NPE and HPE mice. Mitochondria isolated from HPE GP muscles had significantly decreased State 3 and 3_U_ respiration compared to NPE GP when provided with succinate under coupled conditions (Figure 5a and Table S1). HPE GP muscle mitochondrial respiration of CII‐CIV and through CIV is significantly decreased in uncoupled conditions when given pyruvate as substrate (Figure 5b). Thus, in both coupled and uncoupled conditions, HPD reduces respiratory capacity of isolated mitochondria provided with glucose‐derived substrates. Similarly, HPE GP mitochondrial respiration of CII‐CIV is decreased compared to NPE GP when given palmitoyl carnitine (PC) as substrate under uncoupled conditions (Figure 5c). We analyzed mitochondrial protein content in isolated mitochondria samples by western blot to determine if exercise combined with HPD affects oxidative phosphorylation (OXPHOS) proteins. We found no differences in representative OXPHOS protein content across all groups after accounting for protein loading (Figure S3b–e). We performed correlation analysis using Spearman's Rank Correlation test to explore the relationship between low‐volume training and GP mitochondrial respiration. We found no correlation between these variables for the GP EC (*r*_s_ = −0.071, *p* = 0.88), EF (*r*_s_ = 0.57, *p* = 0.18), or FAO (*r*_s_ = −0.29, *p* = 0.53) assays. For liver mitochondria, there is no significant difference between NPE and HPE in coupled or uncoupled conditions when provided either succinate, pyruvate, or PC as substrates (Figure 5d–f and Table S1). These results are consistent with our previous study demonstrating that HPD dysregulates skeletal muscle, but not liver, metabolism. Moreover, these data reveal that HPD induces muscle mitochondrial dysfunction despite exercise training.

**FIGURE 5:**
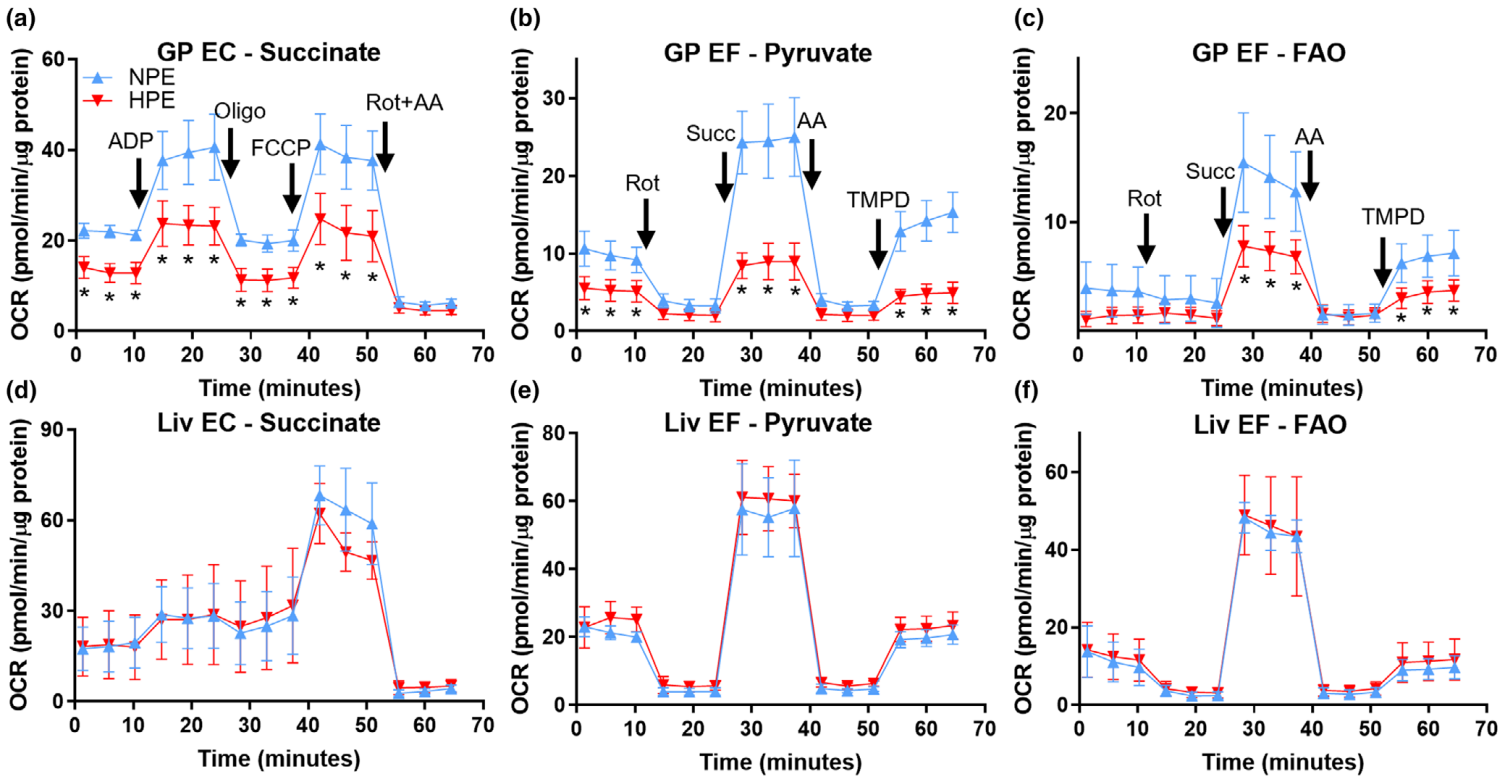
Voluntary exercise training does not prevent high‐phosphate diet‐induced skeletal muscle mitochondrial dysfunction. Coupled respiration of (a) gastrocnemius plantaris (GP) and (d) liver (liv) isolated mitochondria provided with 10 mM succinate and 2 μM rotenone. Uncoupled respiration of (b) GP and (e) liv isolated mitochondria provided 10 mM pyruvate, 2 mM malate, and 4 μM FCCP. Uncoupled respiration of (c) GP and (f) liv isolated mitochondria provided 60 mM PC, 0.4 mM malate, and 4 μM FCCP. *n* = 4/condition. Data are mean ± SD. *p* determined by unpaired Student's *t*‐test.

### 3.7 Voluntary exercise training induces gene expression changes indicative of skeletal muscle adaptation to exercise

To explore why mitochondrial function, but not exercise duration, is decreased in exercise‐trained HPD‐fed mice, we performed RNA sequencing on GP muscles and compared gene expression across four experimental groups (NPS, NPE, HPS, HPE). In prior studies we extensively investigated the dietary impact on GP muscle gene expression (Peri‐Okonny et al., 2019), so here we focused on how the interaction of diet and exercise affects gene expression. Principal component analysis (PCA) revealed distinct clustering of gene expression solely reflective of diet under sedentary conditions (NPS vs. HPS). Gene expression from exercise‐trained muscle was vastly different from sedentary muscle but was similar in exercised muscle from different dietary groups (NPE vs. HPE) (Figure 6a). We calculated differential gene expression (DEG) between NPS and NPE, HPS and HPE, NPS and HPS, and NPE and HPE. Heatmaps display clear downregulation and upregulation patterns of gene expression in muscle from NPE and HPE mice (Figure 6b,c). The total number of DEGs between NPS and NPE mice was 9467: 4927 genes were significantly downregulated, and 4540 genes were significantly upregulated with exercise training (Table S2). The total number of DEGs between HPS and HPE mice was 8009: 4126 genes were significantly downregulated, and 3883 genes were significantly upregulated with exercise training (Table S3). Moreover, we identified specific genes that were uniquely regulated by diet in the setting of exercise: 1742 unique genes were downregulated in NPS versus NPE compared to 941 unique genes downregulated in HPS versus HPE muscle (Table S4). We also identified 1117 unique genes that were upregulated in NPS versus NPE compared to 756 upregulated in HPS versus HPE (Table S5). Surprisingly, we detected only 141 genes commonly downregulated in both comparisons (NPS vs. NPE and HPS vs. HPE) (Table S6), whereas 2979 genes were commonly upregulated in both comparisons (Table S7).

**FIGURE 6:**
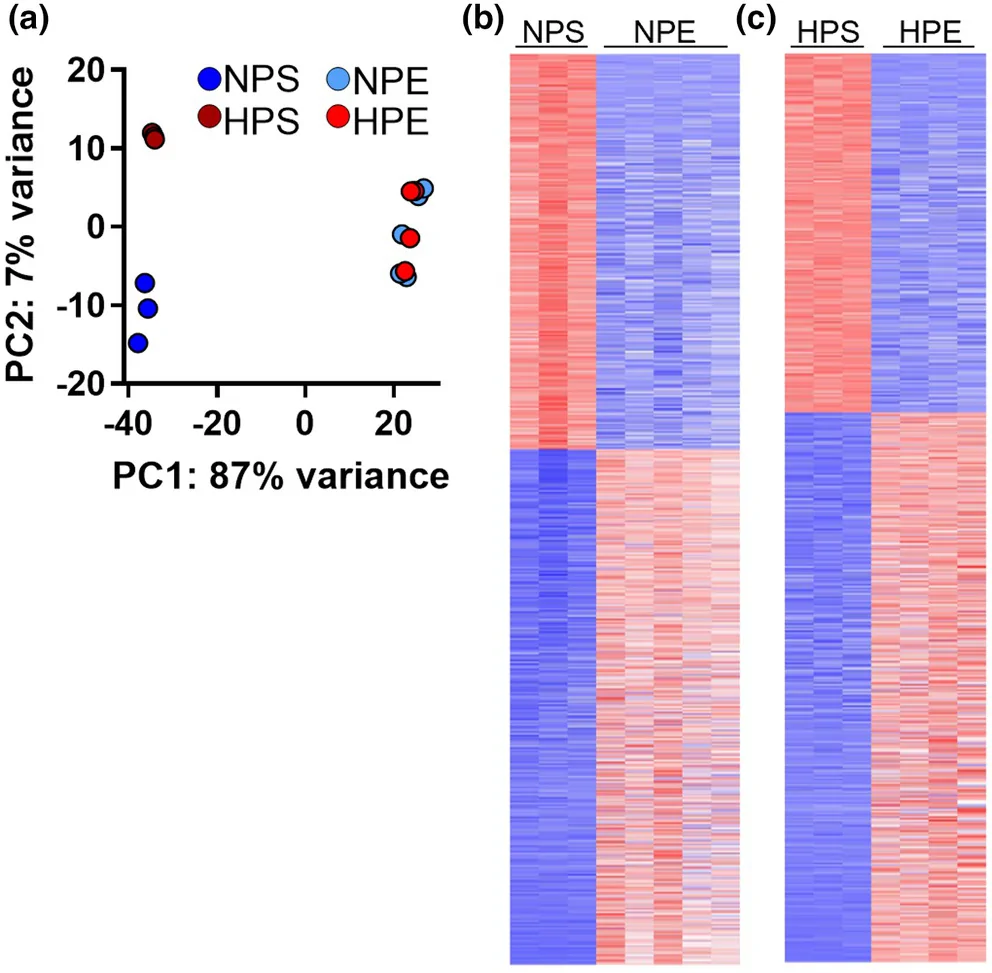
Exercise training alters muscle gene expression regardless of phosphate consumption. (a) Principal component analysis of RNA sequencing of muscle. (b) Heatmaps of DEGs between NPS and NPE and (c) between HPS and HPE muscle. *n* = 3–5 mice/condition.

We performed Reactome Pathway analysis to gain mechanistic insight into how exercise and diet alter gene expression in skeletal muscle. Among the top pathways regulated by exercise training in muscle, regardless of dietary regimen, were metabolism of proteins, metabolism, translation, aerobic respiration, and signal transduction, to name a few (Figure 7a,b). Because changes in directionality of gene expression provide additional mechanistic clarity, we performed Reactome Pathway analysis of DEGs only upregulated in NPS versus NPE and upregulated in HPS versus HPE. The top categories of DEGs that were commonly upregulated between these groups were translation, aerobic respiration, metabolism of proteins, metabolism and others, all of which are indicative of skeletal muscle adaptation to exercise training (Hood & Saleem, 2007; Jensen et al., 2011; Leal et al., 2018; Plotkin et al., 2021) (Figure 7c) and reflect categories of DEGs independent of directionality (Figure 7a,b). We generated a Venn diagram to get a perspective of DEGs across four different comparisons of experimental groups. Many DEGs were shared between NPS versus NPE and HPS versus HPE, as discussed in detail above. DEGs commonly regulated in NPS versus HPS were also differentially expressed in NPS versus NPE and HPS versus HPE. Consistent with PCA, very few genes (only 10) were differentially expressed between NPE and HPE (Figure 6d and Table S8).

**FIGURE 7:**
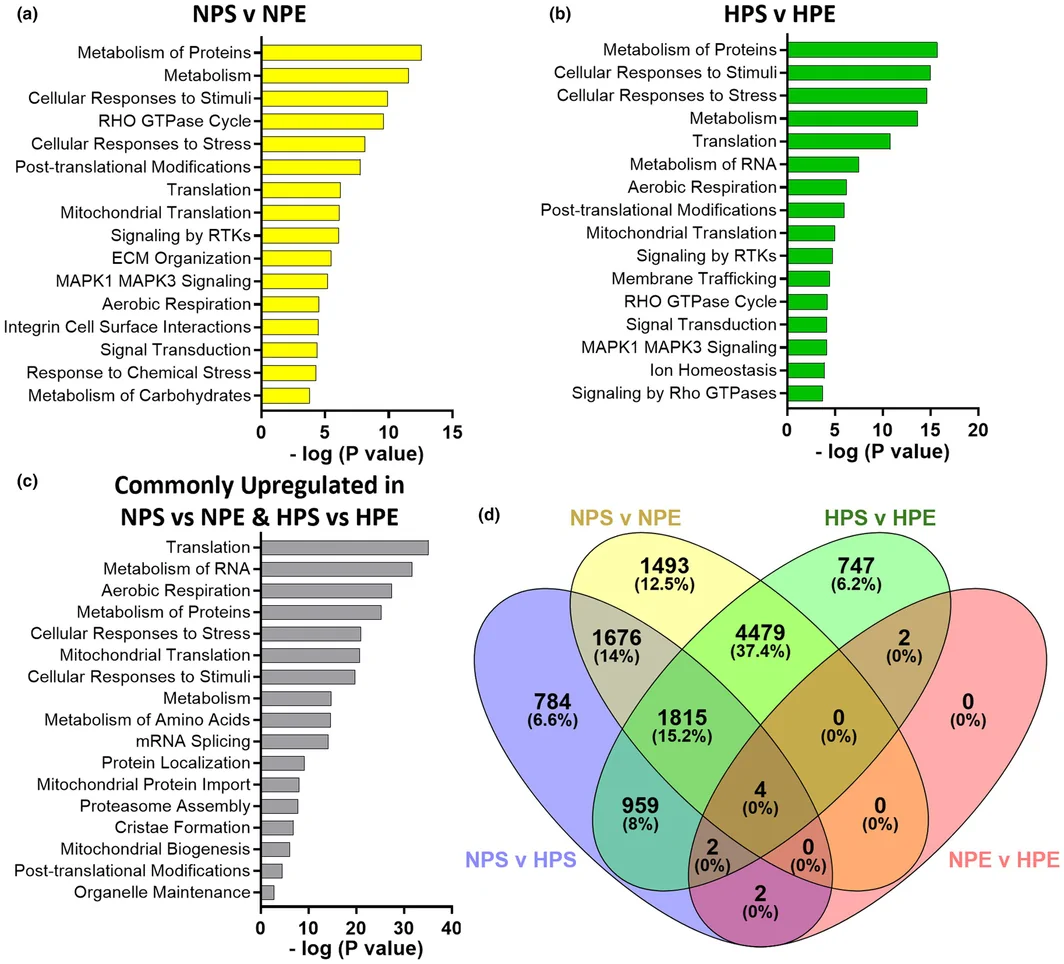
Exercise mitigates dysregulation of subsets of high phosphate‐induced muscle gene expression. (a) Reactome Pathway analysis of DEGs between NPS and NPE and (b) between HPS and HPE. (c) Reactome Pathway analysis of commonly upregulated genes in NPS versus NPE compared to HPS versus HPE. (d) Venn diagram of commonly regulated DEGs between experimental groups. *n* = 3–5 mice/condition.

### 3.8 Short‐term HPD consumption partially counteracts beneficial exercise‐induced gene expression

Reactome Pathway analysis was not performed on 141 genes that were commonly downregulated in NPS versus NPE and downregulated in HPS versus HPE as this analysis is neither robust nor yields significant results given the small number of DEGs. Instead, we analyzed DEGs uniquely regulated between these groups. We uncovered enrichment of several key signal transduction and immune Reactome Pathways when analyzing uniquely downregulated genes in NPS versus NPE muscle (Figure 8a), and enrichment of many of the same Reactome Pathways when analyzing uniquely upregulated genes in HPS versus HPE muscle (Figure 8b). Genes upregulated in HPS versus HPE muscle involved in immune system function include prolylcarboxypeptidase (*Prcp*), a pro‐inflammatory protease, and placenta associated 8 (*Plac8*), which regulates autophagic processes. Other upregulated genes are involved in pro‐inflammatory cytokine signaling, including tumor necrosis factor (*Tnf*), TRAF‐interacting protein with FHA domain (*Tifa*), interleukin 12 subunit beta (*Il12b*), tumor necrosis factor superfamily member 8 (*Tnfsf8*), and signal transducer and activator of transcription 4 (*Stat4*) (Figure 7e). These results suggest that transient pro‐inflammatory signaling responses that typically occur after exercise to aid in muscle repair (Langston & Mathis, 2024) may instead persist when HPD is consumed.

**FIGURE 8:**
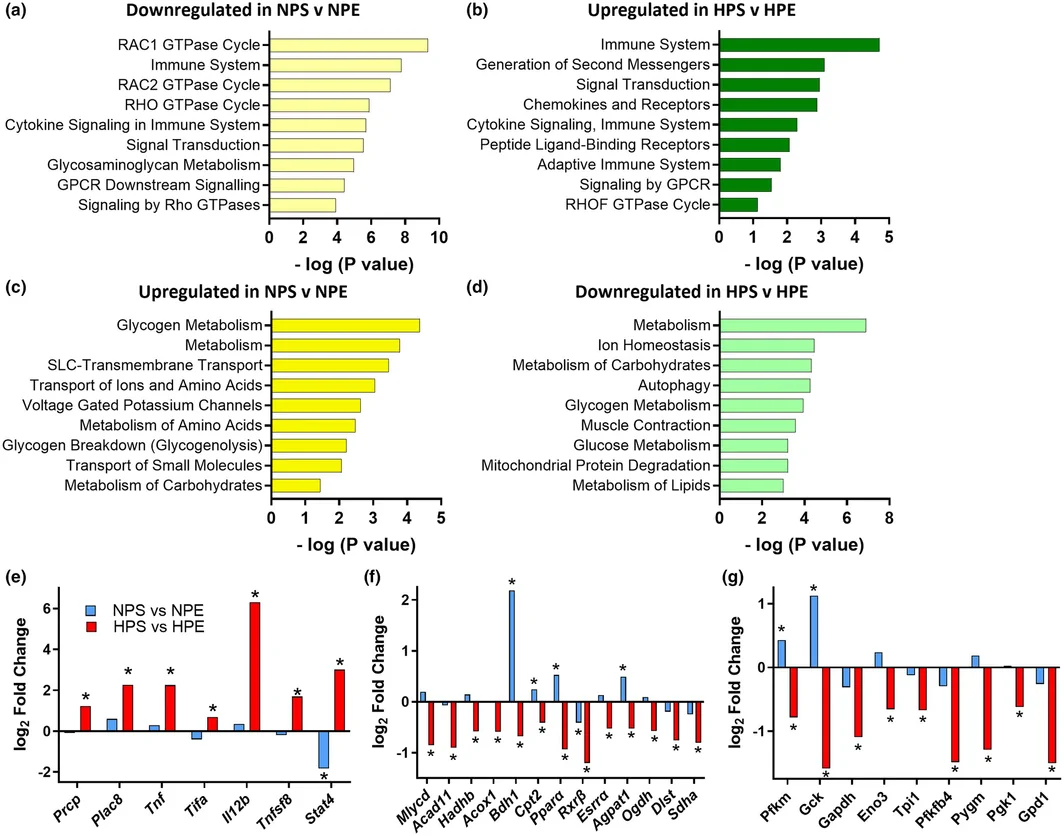
Exercise differentially impacts muscle gene expression in trained mice compared to sedentary mice consuming different diets. Reactome pathway analysis of (a) uniquely downregulated genes (light yellow) in NPS versus NPE compared to HPS versus HPE, (b) uniquely upregulated genes (dark green) in HPS versus HPE compared to NPS versus NPE, (c) uniquely upregulated genes (dark yellow) in NPS versus NPE compared to HPS versus HPE and (d) uniquely downregulated genes (light green) in HPS versus HPE compared to NPS versus NPE. (e) Expression of selected immune system‐related genes, (f) fatty‐acid metabolism‐related genes, and (g) glucose metabolism‐related genes differentially regulated in NPS versus NPE and HPS versus HPE. *p_adj_ <0.05 of NPS versus NPE or HPS versus HPE, determined by Wald test and Benjamini‐Hochberg FDR.

Because exercise training metabolically remodels skeletal muscle (Handy & Holloway, 2023; Hargreaves & Spriet, 2020; Kelahmetoglu et al., 2020; McGee & Hargreaves, 2020), we were not surprised that metabolism genes were differentially expressed between sedentary and exercised muscle. However, we were intrigued to discover enrichment of many metabolism‐related Reactome Pathways when analyzing uniquely upregulated genes in NPS versus NPE muscle and uniquely downregulated genes in HPS versus HPE muscle (Figure 8c,d). Fatty‐acid metabolism‐related genes that were upregulated in NPS versus HPS (Peri‐Okonny et al., 2019) and in NPS versus NPE were conversely downregulated in HPS versus HPE and include malonyl‐CoA decarboxylase (*Mlycd*), acyl‐CoA dehydrogenase family member 11 (*Acad11*), hydroxyacyl‐CoA dehydrogenase trifunctional multienzyme complex subunit beta (*Hadhb*), acyl‐CoA oxidase 1 (*Acox1*), and 3‐hydroxybutyrate dehydrogenase 1 (*Bdh1*) (Figure 8f). Importantly, carnitine‐palmitoyl transferase 2 (*Cpt2*), the inner mitochondrial membrane transporter for fatty‐acids, was downregulated in HPS versus HPE muscle (Figure 8f). Genes involved in lipid signaling and transcriptional regulators of lipid metabolism were also decreased in HPS versus HPE compared to NPS versus NPE muscle, including peroxisome proliferator activated receptor alpha (*Pparα*), retinoid X receptor beta (*Rxrβ*), and estrogen related receptor alpha (*Esrrα*) (Figure 8f). Downregulation of 1‐acylglycerol‐3‐phosphate O‐acyltransferase 1 (*Agpat1*) suggests that triacylglycerol and phospholipid synthesis is dysregulated in HPS versus HPE muscle (Figure 8f). Oxoglutarate dehydrogenase (*Ogdh*) and dihydrolipoamide S‐succinyltransferase (*Dlst*), both proteins in the α‐ketoglutarate dehydrogenase complex, and succinate dehydrogenase subunit A (*Sdha*) were all downregulated in HPS versus HPE (Figure 8f,g). Reduced expression may mirror decreased protein levels which could impact carbohydrate and fatty‐acid metabolism through diminished Krebs cycle activity. Additionally, reduced expression of *Sdha* may represent decreased protein levels and could disrupt formation of the succinate dehydrogenase complex overall, potentially impacting ETC activity. Intriguingly, glycolysis genes phosphofructokinase (*Pfkm*), glucokinase (*Gck*), glyceraldehyde‐3‐phosphate dehydrogenase (*Gapdh*), enolase‐3 (*Eno3*), triosephosphate isomerase 1 (*Tpi1*), and 6‐phosphofructo‐2‐kinase/fructose‐2,6‐biphosphatase4 (*Pfkfb4*) were downregulated in HPS versus HPE muscle compared to NPS versus NPE muscle (Figure 8g). Other downregulated genes involved in glucose and glycogen metabolism include glycogen phosphorylase (*Pygm*), phosphoglycerate kinase 1 (*Pgk1*), and glycerol‐3‐phosphate dehydrogenase 1 (*Gpd1*) (Figure 8g). Collectively, these data reveal that skeletal muscle transcriptome of HPD‐fed mice partially adapts to exercise training, but HPD prevents complete restoration of metabolic gene expression patterns observed in muscle of NPS versus NPE mice.

## 4 DISCUSSION

Metabolic disease is increasing globally and correlates with elevated consumption of ultra‐processed foods (Canhada et al., 2023; Godsey et al., 2025; Moore et al., 2017; Zhang et al., 2024). Physical inactivity is prevalent, further contributing to metabolic syndrome (Kerr & Booth, 2022), collectively elevating mortality risk and warranting intervention (Martinez‐Gomez et al., 2024; Shi et al., 2020). We previously demonstrated that 12 weeks of HPD reduces exercise capacity in mice through dysregulated skeletal muscle substrate utilization and mitochondrial dysfunction (Peri‐Okonny et al., 2019). Here, we uncovered that Pi loading occurs within 1 day of HPD consumption, and only 5 weeks of HPD is sufficient to impair skeletal muscle mitochondrial function.

Exercise is an effective treatment for metabolic syndrome by reducing the risk of mortality and promoting whole‐body health. Voluntary wheel running in mice protects against toxic protein aggregation in Parkinson's disease and improves muscle function in muscular dystrophy models (Bittel et al., 2024; Hamm et al., 2021; Manta et al., 2019; Zhou et al., 2017), and it aligns with mouse behavior, making it a low‐commitment high‐reward model to experimentally introduce exercise (Poole et al., 2020). We investigated whether exercise training could be used as a lifestyle intervention to restore skeletal muscle metabolism in the context of HPD. We introduced voluntary wheel running in mice after 5 weeks on the diet when skeletal muscle mitochondrial function was already impaired. We did not detect any significant differences in the average distance run per day or average daily distance run per week between trained NPD‐ and HPD‐fed mice throughout the study. As commonly reported, we observed variability in distance run per day and week within each group; however, body composition measurements reflect effective exercise training responses for all mice, regardless of individual exercise training volume. Additionally, exploratory correlation analysis using Spearman's Rank Correlation revealed no relationship between low training volume and mitochondrial respiration, indicating minimal distance run per day does not influence low OCR of HPE GP isolated mitochondria.

It was surprising that exercise‐trained HPD‐fed mice performed similarly to trained NPD‐fed mice in forced exercise tests yet displayed mitochondrial dysfunction. These results imply that the detrimental effects of HPD cannot be reversed with voluntary exercise training. It is important to note that low respiration does not always indicate mitochondrial dysfunction, as reduced respiration can be an adaptive response to changing physiological demands. However, decreased exercise capacity and dysregulated metabolic gene expression in sedentary HPD‐fed mice, which remain altered in trained mice, suggest mitochondrial dysfunction is more likely. Exercise can induce mitochondrial biogenesis (Hood & Saleem, 2007), ultimately overcoming dysfunction and restoring or improving exercise duration. Indeed, we observed increased mitochondrial content with exercise training regardless of diet, indicative of mitochondrial biogenesis. Western blot analysis of isolated mitochondria revealed no significant differences in representative OXPHOS proteins between NPE and HPE mice. These results suggest that mitochondrial content does not account for sustained mitochondrial dysfunction despite rescued exercise duration.

Although HPD directly impacts exercise, there may also be compensatory mechanisms that support exercise performance despite reduced skeletal muscle mitochondrial function. Exercise training increases stiffness of the muscle‐extracellular matrix‐tendon unit, which enhances the body's ability to store and use elastic energy more efficiently (Arampatzis et al., 2006; Hughes et al., 2018). Trained individuals may have neural adaptations that increase muscle coactivation, similarly facilitating more efficient use of elastic energy (Heise et al., 2008; Hughes et al., 2018). Exercise also remodels the cardiovascular system for improved cardiac output, increasing aerobic power and endurance performance (Hellsten & Nyberg, 2015). Moreover, we cannot rule out the possibility that HPD attenuates the training response. Whether these processes or other molecular changes are involved in sustained exercise performance of trained HPD‐fed mice remains to be determined. An intriguing outcome of this study is the tissue specificity of HPD‐induced mitochondrial dysfunction. In contrast to muscle, preservation of liver mitochondrial function when challenged by HPD suggests that not all highly metabolic tissues are equally influenced by diet. The mechanisms responsible for muscle‐specific HPD‐induced metabolic dysregulation will be explored in future work.

We explored possible mechanisms of mitochondrial dysfunction using RNA sequencing. In previous studies we reported that HPD remodels metabolic gene expression in skeletal muscle (Peri‐Okonny et al., 2019). Exercise‐trained GP muscle showed upregulation of carbohydrate and fatty‐acid metabolism related genes as well as genes involved in mitochondrial biogenesis, translation, and cristae formation, regardless of diet. These changes are consistent with published skeletal muscle adaptation to exercise; exercise increases FAO, oxidative metabolism, glucose transport, and improves insulin sensitivity (Jensen et al., 2011; Leal et al., 2018; Plotkin et al., 2021; Smith et al., 2023; Zoll et al., 2002). However, exercise training does not completely restore DEG patterns in HPS versus HPE to those observed in NPS versus NPE. Genes involved in β‐oxidation and fatty‐acid signaling were downregulated in muscle of HPE compared to HPS mice, similar to what we observed in HPS compared to NPS muscle. Intriguingly, glycolysis and glycogen metabolism‐related genes that were upregulated in HPS compared to NPS are downregulated in HPE compared to HPS muscle. This suggests that there may be a regulatory battle at the transcriptional level: in response to exercise, muscle attempts to restore substrate utilization by increasing expression of fatty‐acid metabolism related genes and decreasing expression of glucose metabolism related genes, but HPD prevents complete rescue of gene expression. This could be one reason why we detected sustained mitochondrial dysfunction in HPE muscle, as decreased expression of CPT2, β‐oxidation genes, and other lipid‐regulated genes may reflect decreased protein levels which could reduce fatty acid utilization. Simultaneous decreased expression of some glucose metabolism related genes and protein levels could also diminish glucose utilization, but direct glycolytic functional measurements are necessary to confirm altered glucose usage. Ultimately, exercise training in the presence of HPD induces some of the beneficial metabolic gene expression changes observed when comparing muscle from NPS versus NPE mice, but HPD‐induced dysregulation persists.

Interestingly, comparing DEGs in HPS versus HPE mice to DEGs in NPS versus NPE mice reveals unique inflammatory pathway gene signatures. Reactome pathway analysis reveals that immune system, adaptive immune system, and cytokine signaling genes are increased in HPE muscle, while these pathways are downregulated in NPE muscle. Importantly, these same genes are also downregulated in HPS muscle, so upregulation of inflammatory gene expression is a unique feature of HPD and exercise training combined. Exercise induces lipolysis of both intramuscular lipid stores and adipose tissue (Mulla et al., 2000; Zhu & Guo, 2025), implying that circulating free fatty acids may increase. Fatty‐acid binding proteins are among the genes upregulated in HPE, and if also reflected at the protein level, suggest that skeletal muscle in these mice might be capable of enhanced fatty‐acid uptake. Though we have not measured lipid content in serum or muscle in HPD‐fed trained mice, downregulation of β‐oxidation genes reflected in protein expression may increase levels of incompletely oxidized fatty acids and fatty acid derivatives. In obesity and overnutrition models, dysregulation of fatty acid transport and intramyocellular lipid and lipid derivative accumulation contributes to lipotoxicity and compromises insulin signaling in the muscle (Eckardt et al., 2011; Montgomery et al., 2019; Sylow et al., 2021). Insulin resistance attenuates PI3K‐Akt signaling, activating inflammatory markers via JNK and IKKB. Inflammation and increased substrate availability can induce reactive oxygen species (ROS) production in mitochondria (de Luca & Olefsky, 2008; Wu & Ballantyne, 2017). Therefore, increased expression of inflammatory genes in HPS versus HPE muscle may be an adaptive response of muscle to elevation of partially oxidized fatty acids. Indeed, we observed decreased fatty‐acid metabolism gene expression, which could foster continued mitochondrial dysfunction. Future studies will explore these mechanistic possibilities.

While voluntary exercise training may rescue exercise endurance, sustained reduction of mitochondrial respiratory capacity is a major concern. Mitochondrial dysfunction is indicative of disrupted electron transfer in the ETC, which can cause electron accumulation and ROS formation (Handy & Holloway, 2023; Powers et al., 2011). Chronic ROS accumulation promotes protein degradation, and if left unchecked, can lead to muscle atrophy, which is associated with aging (Grevendonk et al., 2021; Hyatt et al., 2019; Powers et al., 2011). We have shown that HPD impairs mitochondrial function through a mechanism that is not completely reversible with exercise alone. Phosphate restriction is recommended for CKD patients to relieve the filtration burden on the kidneys and reduce the risk for CKD associated pathologies (Biruete et al., 2023). It may be that limiting dietary Pi intake in conjunction with physical exercise could alleviate decreased mitochondrial respiratory capacity, preventing damage that chronic mitochondrial dysfunction can induce through ROS, but these mechanisms require further investigation.

Here we tested whether voluntary exercise mitigates HPD‐induced metabolic dysregulation in skeletal muscle without dietary intervention. However, our results imply that this strategy is not sufficient to restore muscle metabolism, and that it may be the case that exercise training and dietary intervention are required. Whether introducing wheel running at the onset of diet could prevent mitochondrial dysfunction and maintain exercise endurance and metabolic gene expression remains to be investigated. Additionally, whether other forms of exercise training could prevent HPD‐induced skeletal muscle metabolic dysregulation remains to be investigated. Ultimately, our work highlights Pi as a target for regulating skeletal muscle metabolism and function and provides additional support that skeletal muscle is an unrecognized tissue that modulates Pi homeostasis.

## AUTHOR CONTRIBUTIONS

**Kedryn K. Baskin:** Conceptualization; data curation; formal analysis; funding acquisition; investigation; methodology; project administration; resources; supervision; visualization. **Hannah F. Sanders:** Data curation; formal analysis; funding acquisition; investigation; methodology; visualization. **Dominic W. Kolonay:** Data curation; formal analysis; funding acquisition; investigation; methodology; visualization. **Jade A. Blackwell:** Data curation; formal analysis; investigation; methodology. **Jiaru Liu:** Data curation; formal analysis; investigation; methodology. **Isabella R. Dobrski:** Data curation; formal analysis; investigation; methodology. **Daniel A. Branch:** Data curation; formal analysis; investigation; methodology. **Lucas A. Lefers:** Data curation; formal analysis; investigation; methodology. **Benjamen H. Kern:** Data curation; formal analysis; investigation; methodology.

## FUNDING INFORMATION

This work was supported in part by T32HL134616 (to H.F.S. and D.W.K.), T32GM141955 (to B.H.K.), K01DK116916 and R01HL166520 (to K.K.B.).

## ETHICS STATEMENT

All procedures were approved by The Ohio State University Institutional Animal Care and Use Committee and comply with the Physiological Reports' Author Guidelines on ethics.

## Supporting information


**Figure S1:** Scheme of study design. (a) 22‐week‐old male mice were placed on normal‐phosphate diet (0.6% Pi) and high‐phosphate diet (1.2% Pi) for 14 weeks. Voluntary exercise training was introduced 5 weeks after diet initiation. (b) Scheme of serum, urine, and feces collection throughout the study duration (D; day, W; week).

**Figure S2:** Liver (liv) mitochondrial function is not affected after 5 weeks of high‐phosphate diet (HPD) consumption. (a) Coupled respiration of isolated liv mitochondria provided with 10 mM succinate and 2 μM rotenone. (b) Uncoupled respiration of isolated liv mitochondria provided with 10 mM pyruvate, 2 mM malate, and 4 μM FCCP. *n* = 2/condition. Data are mean ± SD.

**Figure S3:** Mitochondrial content is increased in trained GP but oxidative phosphorylation (OXPHOS) protein abundance within GP mitochondria is not significantly altered by diet or exercise. (a) qPCR of mitochondrial and nuclear DNA from GP muscle. *n* = 3–4/condition. Data are mean ± SD. *p* determined by one‐way ANOVA. (b) Short exposure and (c) longer exposure of entire western blot membrane detecting representative OXPHOS proteins in 5 μg of isolated mitochondria. (d) Total mitochondrial protein loaded visualized by Ponceau stain. (e) Combined quantification of representative OXPHOS proteins from all samples. Representative images shown as *n* = 3/condition and quantification of *n* = 6/condition. Data are mean ± SD.

**Table S1:** State 3 U respiration, State 4 respiration, and RCR values.

**Table S2:** Differentially expressed genes (NPS vs. NPE; *p*_adj_ <0.05 and |[log2FC]| >0.5).

**Table S3:** Differentially expressed genes (HPS vs. HPE; *p*_adj_ <0.05 and |[log2FC]| >0.5).

**Table S4:** Decreased expression of genes with exercise, dependent on diet.

**Table S5:** Increased expression of genes with exercise, dependent on diet.

**Table S6:** Decreased expression of common genes with exercise, independent of diet.

**Table S7:** Increased expression of common genes with exercise, independent of diet.

**Table S8:** Differentially expressed genes (NPE vs. HPE; *p*_adj_ <0.05 and |[log2FC]| >0.5).

## Data Availability

RNA sequencing data generated in this study have been deposited in the Gene Expression Omnibus under accession number GSE325957. Previously published RNA sequencing data used in this study can be obtained under accession number GSE120958.

## References

[phy271139-bib-0001] Ahn, C. , Hevener, A. L. , Goodyear, L. J. , Bodine, S. C. , Esser, K. A. , Seldin, M. M. , & Sparks, L. M. (2025). Exercise training remodels inter‐organ endocrine networks. Molecular Metabolism, 99, 102219. 10.1016/j.molmet.2025.102219 PMC12340562 40701293

[phy271139-bib-0002] Ahn, C. , Zhang, T. , Rode, T. , Yang, G. , Chugh, O. K. , Ellis, S. , Ghayur, S. , Mehta, S. , Salzman, R. , Jiang, H. , Parker, S. C. J. , Burant, C. F. , & Horowitz, J. F. (2025). Molecular responses in abdominal subcutaneous adipose tissue after a session of endurance exercise: Effects of exercise intensity. The Journal of Physiology, 603, 6895–6919. 10.1113/JP289339 PMC12645526 41076654

[phy271139-bib-0003] Arampatzis, A. , de Monte, G. , Karamanidis, K. , Morey‐Klapsing, G. , Stafilidis, S. , & Bruggemann, G. P. (2006). Influence of the muscle‐tendon unit's mechanical and morphological properties on running economy. Journal of Experimental Biology, 209, 3345–3357. https://www.ncbi.nlm.nih.gov/pubmed/16916971 10.1242/jeb.02340 16916971

[phy271139-bib-0004] Ashcroft, S. P. , Stocks, B. , Egan, B. , & Zierath, J. R. (2024). Exercise induces tissue‐specific adaptations to enhance cardiometabolic health. Cell Metabolism, 36, 278–300. 10.1016/j.cmet.2023.12.008 38183980

[phy271139-bib-0005] Biruete, A. , Hill Gallant, K. M. , Lloyd, L. , Meade, A. , Moe, S. M. , St‐Jules, D. E. , & Kistler, B. M. (2023). Phostering a clear message: The evolution of dietary phosphorus Management in Chronic Kidney Disease. Journal of Renal Nutrition, 33, S13–S20. 10.1053/j.jrn.2023.05.004 PMC10728341 37343779

[phy271139-bib-0006] Bittel, A. J. , Bittel, D. C. , Gordish‐Dressman, H. , & Chen, Y. W. (2024). Voluntary wheel running improves molecular and functional deficits in a murine model of facioscapulohumeral muscular dystrophy. iScience, 27, 108632. 10.1016/j.isci.2023.108632 PMC10770537 38188524

[phy271139-bib-0007] Bradford, M. M. (1976). A rapid and sensitive method for the quantitation of microgram quantities of protein utilizing the principle of protein‐dye binding. Analytical Biochemistry, 72, 248–254. 10.1016/0003-2697(76)90527-3 942051

[phy271139-bib-0008] Calvo, M. S. , Moshfegh, A. J. , & Tucker, K. L. (2014). Assessing the health impact of phosphorus in the food supply: Issues and considerations. Advances in Nutrition, 5, 104–113. 10.3945/an.113.004861 PMC3884091 24425729

[phy271139-bib-0009] Calvo, M. S. , & Uribarri, J. (2013). Public health impact of dietary phosphorus excess on bone and cardiovascular health in the general population. The American Journal of Clinical Nutrition, 98, 6–15. 10.3945/ajcn.112.053934 23719553

[phy271139-bib-0010] Canhada, S. L. , Vigo, A. , Luft, V. C. , Levy, R. B. , Alvim Matos, S. M. , Del Carmen Molina, M. , Giatti, L. , Barreto, S. , Duncan, B. B. , & Schmidt, M. I. (2023). Ultra‐processed food consumption and increased risk of metabolic syndrome in adults: The ELSA‐Brasil. Diabetes Care, 46, 369–376. 10.2337/dc22-1505 PMC9887627 36516280

[phy271139-bib-0011] Cartee, G. D. , Hepple, R. T. , Bamman, M. M. , & Zierath, J. R. (2016). Exercise promotes healthy aging of skeletal muscle. Cell Metabolism, 23, 1034–1047. 10.1016/j.cmet.2016.05.007 PMC5045036 27304505

[phy271139-bib-0012] de Luca, C. , & Olefsky, J. M. (2008). Inflammation and insulin resistance. FEBS Letters, 582, 97–105. 10.1016/j.febslet.2007.11.057 PMC2246086 18053812

[phy271139-bib-0013] Eckardt, K. , Taube, A. , & Eckel, J. (2011). Obesity‐associated insulin resistance in skeletal muscle: Role of lipid accumulation and physical inactivity. Reviews in Endocrine & Metabolic Disorders, 12, 163–172. 10.1007/s11154-011-9168-2 21336841

[phy271139-bib-0014] Garritson, J. D. , & Boudina, S. (2021). The effects of exercise on White and Brown adipose tissue cellularity, metabolic activity and remodeling. Frontiers in Physiology, 12, 772894. 10.3389/fphys.2021.772894 PMC8593176 34795599

[phy271139-bib-0015] Godsey, T. J. , Eden, T. , & Emerson, S. R. (2025). Ultra‐processed foods and metabolic dysfunction: A narrative review of dietary processing, behavioral drivers and chronic disease risk. Metabolites, 15, 784. 10.3390/metabo15120784 PMC12734455 41441026

[phy271139-bib-0016] Grevendonk, L. , Connell, N. J. , McCrum, C. , Fealy, C. E. , Bilet, L. , Bruls, Y. M. H. , Mevenkamp, J. , Schrauwen‐Hinderling, V. B. , Jorgensen, J. A. , Moonen‐Kornips, E. , Schaart, G. , Havekes, B. , de Vogel‐van den Bosch, J. , Bragt, M. C. E. , Meijer, K. , Schrauwen, P. , & Hoeks, J. (2021). Impact of aging and exercise on skeletal muscle mitochondrial capacity, energy metabolism, and physical function. Nature Communications, 12, 4773. 10.1038/s41467-021-24956-2 PMC8346468 34362885

[phy271139-bib-0017] Hamm, S. E. , Fathalikhani, D. D. , Bukovec, K. E. , Addington, A. K. , Zhang, H. , Perry, J. B. , McMillan, R. P. , Lawlor, M. W. , Prom, M. J. , Vanden Avond, M. A. , Kumar, S. N. , Coleman, K. E. , Dupont, J. B. , Mack, D. L. , Brown, D. A. , Morris, C. A. , Gonzalez, J. P. , & Grange, R. W. (2021). Voluntary wheel running complements microdystrophin gene therapy to improve muscle function in mdx mice. Molecular Therapy ‐ Methods & Clinical Development, 21, 144–160. 10.1016/j.omtm.2021.02.024 PMC8020351 33850950

[phy271139-bib-0018] Handy, R. M. , & Holloway, G. P. (2023). Insights into the development of insulin resistance: Unraveling the interaction of physical inactivity, lipid metabolism and mitochondrial biology. Frontiers in Physiology, 14, 1151389. 10.3389/fphys.2023.1151389 PMC10157178 37153211

[phy271139-bib-0019] Hargreaves, M. , & Spriet, L. L. (2020). Skeletal muscle energy metabolism during exercise. Nature Metabolism, 2, 817–828. 10.1038/s42255-020-0251-4 32747792

[phy271139-bib-0020] Heise, G. , Shinohara, M. , & Binks, L. (2008). Biarticular leg muscles and links to running economy. International Journal of Sports Medicine, 29, 688–691. https://www.ncbi.nlm.nih.gov/pubmed/18085504 10.1055/s-2007-989372 18085504

[phy271139-bib-0021] Heller, A. , Fischer, L. H. , Wolfbeis, O. S. , & Goepferich, A. (2012). Long time monitoring of the respiratory activity of isolated mitochondria. Experimental Cell Research, 318, 1667–1672. https://www.ncbi.nlm.nih.gov/pubmed/22659171 10.1016/j.yexcr.2012.05.007 22659171

[phy271139-bib-0022] Hellsten, Y. , & Nyberg, M. (2015). Cardiovascular adaptations to exercise training. Comprehensive Physiology, 6, 1–32. https://www.ncbi.nlm.nih.gov/pubmed/26756625 10.1002/cphy.c140080 26756625

[phy271139-bib-0023] Hood, D. A. , & Saleem, A. (2007). Exercise‐induced mitochondrial biogenesis in skeletal muscle. Nutrition, Metabolism, and Cardiovascular Diseases, 17, 332–337. 10.1016/j.numecd.2007.02.009 17467251

[phy271139-bib-0024] Hughes, D. C. , Ellefsen, S. , & Baar, K. (2018. https://www.ncbi.nlm.nih.gov/pubmed/28490537). Adaptations to endurance and strength training. Cold Spring Harbor Perspectives in Medicine, 8, a029769. 10.1101/cshperspect.a029769 PMC5983157 28490537

[phy271139-bib-0025] Hyatt, H. , Deminice, R. , Yoshihara, T. , & Powers, S. K. (2019). Mitochondrial dysfunction induces muscle atrophy during prolonged inactivity: A review of the causes and effects. Archives of Biochemistry and Biophysics, 662, 49–60. 10.1016/j.abb.2018.11.005 PMC6783132 30452895

[phy271139-bib-0026] Jensen, J. , Rustad, P. I. , Kolnes, A. J. , & Lai, Y. C. (2011). The role of skeletal muscle glycogen breakdown for regulation of insulin sensitivity by exercise. Frontiers in Physiology, 2, 112. 10.3389/fphys.2011.00112 PMC3248697 22232606

[phy271139-bib-0027] Kelahmetoglu, Y. , Jannig, P. R. , Cervenka, I. , Koch, L. G. , Britton, S. L. , Zhou, J. , Wang, H. , Robinson, M. M. , Nair, K. S. , & Ruas, J. L. (2020). Comparative analysis of skeletal muscle transcriptional signatures associated with aerobic exercise capacity or response to training in humans and rats. Front Endocrinol (Lausanne), 11, 591476. 10.3389/fendo.2020.591476 PMC7649134 33193103

[phy271139-bib-0028] Kerr, N. R. , & Booth, F. W. (2022). Contributions of physical inactivity and sedentary behavior to metabolic and endocrine diseases. Trends in Endocrinology and Metabolism, 33, 817–827. 10.1016/j.tem.2022.09.002 36283907

[phy271139-bib-0029] Knopfel, T. , Pastor‐Arroyo, E. M. , Schnitzbauer, U. , Kratschmar, D. V. , Odermatt, A. , Pellegrini, G. , Hernando, N. , & Wagner, C. A. (2017). The intestinal phosphate transporter NaPi‐IIb (Slc34a2) is required to protect bone during dietary phosphate restriction. Scientific Reports, 7, 11018. 10.1038/s41598-017-10390-2 PMC5591270 28887454

[phy271139-bib-0030] Kolonay, D. W. , Grueter, C. E. , & Baskin, K. K. (2025). Differential regulation during development, aging, and disease implies heart cell specific functions of the mediator complex. J Mol Cell Cardiol Plus, 12, 100456. https://www.ncbi.nlm.nih.gov/pubmed/40510693 10.1016/j.jmccpl.2025.100456 PMC12162080 40510693

[phy271139-bib-0031] Komuro, A. , Son, B. K. , Nanao‐Hamai, M. , Song, Z. , Ogawa, S. , & Akishita, M. (2024). Effects of a high‐phosphate diet on vascular calcification and abdominal aortic aneurysm in mice. Geriatrics & Gerontology International, 24, 973–981. 10.1111/ggi.14959 39139097

[phy271139-bib-0032] Langston, P. K. , & Mathis, D. (2024). Immunological regulation of skeletal muscle adaptation to exercise. Cell Metabolism, 36, 1175–1183. 10.1016/j.cmet.2024.04.001 PMC11153001 38670108

[phy271139-bib-0033] Leal, L. G. , Lopes, M. A. , & Batista, M. L., Jr. (2018). Physical exercise‐induced myokines and muscle‐adipose tissue crosstalk: A review of current knowledge and the implications for health and metabolic diseases. Frontiers in Physiology, 9, 1307. 10.3389/fphys.2018.01307 PMC6166321 30319436

[phy271139-bib-0034] Lee, A. W. , & Cho, S. S. (2015). Association between phosphorus intake and bone health in the NHANES population. Nutrition Journal, 14, 28. 10.1186/s12937-015-0017-0 PMC4389665 25856461

[phy271139-bib-0035] Manta, A. , Stouth, D. W. , Xhuti, D. , Chi, L. , Rebalka, I. A. , Kalmar, J. M. , Hawke, T. J. , & Ljubicic, V. (2019). Chronic exercise mitigates disease mechanisms and improves muscle function in myotonic dystrophy type 1 mice. The Journal of Physiology, 597, 1361–1381. 10.1113/JP277123 PMC6395422 30628727

[phy271139-bib-0036] Martinez‐Gomez, D. , Luo, M. , Huang, Y. , Rodriguez‐Artalejo, F. , Ekelund, U. , Sotos‐Prieto, M. , Ding, D. , Lao, X. Q. , & Cabanas‐Sanchez, V. (2024). Physical activity and all‐cause mortality by age in 4 multinational Megacohorts. JAMA Network Open, 7, e2446802. 10.1001/jamanetworkopen.2024.46802 PMC11582934 39570587

[phy271139-bib-0037] McGee, S. L. , & Hargreaves, M. (2020). Exercise adaptations: Molecular mechanisms and potential targets for therapeutic benefit. Nature Reviews Endocrinology, 16, 495–505. 10.1038/s41574-020-0377-1 32632275

[phy271139-bib-0038] McKie, G. L. , & Wright, D. C. (2020). Biochemical adaptations in white adipose tissue following aerobic exercise: From mitochondrial biogenesis to browning. Biochemical Journal, 477, 1061–1081. 10.1042/BCJ20190466 32187350

[phy271139-bib-0039] Montgomery, M. K. , de Nardo, W. , & Watt, M. J. (2019). Impact of lipotoxicity on tissue “cross talk” and metabolic regulation. Physiology (Bethesda), 34, 134–149. 10.1152/physiol.00037.2018 30724128

[phy271139-bib-0040] Montgomery, M. K. , de Nardo, W. , & Watt, M. J. (2023). Exercise training induces depot‐specific remodeling of protein secretion in skeletal muscle and adipose tissue of obese male mice. American Journal of Physiology. Endocrinology and Metabolism, 325, E227–E238. 10.1152/ajpendo.00178.2023 37493472

[phy271139-bib-0041] Moore, J. X. , Chaudhary, N. , & Akinyemiju, T. (2017). Metabolic syndrome prevalence by race/ethnicity and sex in the United States, National Health and nutrition examination survey, 1988–2012. Preventing Chronic Disease, 14, E24. 10.5888/pcd14.160287 PMC5364735 28301314

[phy271139-bib-0042] Mulla, N. A. , Simonsen, L. , & Bulow, J. (2000). Post‐exercise adipose tissue and skeletal muscle lipid metabolism in humans: The effects of exercise intensity. The Journal of Physiology, 524(3), 919–928. 10.1111/j.1469-7793.2000.00919.x PMC2269892 10790168

[phy271139-bib-0043] Murphy, R. M. , Watt, M. J. , & Febbraio, M. A. (2020). Metabolic communication during exercise. Nature Metabolism, 2, 805–816. 10.1038/s42255-020-0258-x 32747791

[phy271139-bib-0044] Nigro, P. , Vamvini, M. , Yang, J. , Caputo, T. , Ho, L. L. , Carbone, N. P. , Papadopoulos, D. , Conlin, R. , He, J. , Hirshman, M. F. , White, J. D. , Robidoux, J. , Hickner, R. C. , Nielsen, S. , Pedersen, B. K. , Kellis, M. , Middelbeek, R. J. W. , & Goodyear, L. J. (2023). Exercise training remodels inguinal white adipose tissue through adaptations in innervation, vascularization, and the extracellular matrix. Cell Reports, 42, 112392. 10.1016/j.celrep.2023.112392 PMC10374102 37058410

[phy271139-bib-0045] Oda, N. , Sugihara, K. , Uebanso, T. , Ohminami, H. , Ohnishi, K. , Masuda, M. , Yamanaka‐Okumura, H. , & Taketani, Y. (2023). Dietary phosphate disturbs of gut microbiome in mice. Journal of Clinical Biochemistry and Nutrition, 73, 221–227. 10.3164/jcbn.23-9 PMC10636580 37970557

[phy271139-bib-0046] Oliveros, J. C. , & Venny, C. An interactive tool for comparing lists with Venn's diagrams. https://bioinfogp.cnb.csic.es/tools/venny/index.html

[phy271139-bib-0047] Peri‐Okonny, P. , Baskin, K. K. , Iwamoto, G. , Mitchell, J. H. , Smith, S. A. , Kim, H. K. , Szweda, L. I. , Bassel‐Duby, R. , Fujikawa, T. , Castorena, C. M. , Richardson, J. , Shelton, J. M. , Ayers, C. , Berry, J. D. , Malladi, V. S. , Hu, M. C. , Moe, O. W. , Scherer, P. E. , & Vongpatanasin, W. (2019). High‐phosphate diet induces exercise intolerance and impairs fatty acid metabolism in mice. Circulation, 139, 1422–1434. 10.1161/CIRCULATIONAHA.118.037550 PMC6411426 30612451

[phy271139-bib-0048] Plotkin, D. L. , Roberts, M. D. , Haun, C. T. , & Schoenfeld, B. J. (2021). Muscle fiber type transitions with exercise training: Shifting perspectives. Sports (Basel), 9, 127. 10.3390/sports9090127 PMC8473039 34564332

[phy271139-bib-0049] Poole, D. C. , Copp, S. W. , Colburn, T. D. , Craig, J. C. , Allen, D. L. , Sturek, M. , O'Leary, D. S. , Zucker, I. H. , & Musch, T. I. (2020). Guidelines for animal exercise and training protocols for cardiovascular studies. American Journal of Physiology. Heart and Circulatory Physiology, 318, H1100–H1138. 10.1152/ajpheart.00697.2019 PMC7254566 32196357

[phy271139-bib-0050] Powers, S. K. , Ji, L. L. , Kavazis, A. N. , & Jackson, M. J. (2011). Reactive oxygen species: Impact on skeletal muscle. Compr Physiol, 1, 941–969. 10.1002/j.2040-4603.2011.tb00348.x PMC3893116 23737208

[phy271139-bib-0051] Quiros, P. M. , Goyal, A. , Jha, P. , & Auwerx, J. (2017). Analysis of mtDNA/nDNA ratio in mice. Current Protocols in Mouse Biology, 7, 47–54. https://www.ncbi.nlm.nih.gov/pubmed/28252199 10.1002/cpmo.21 PMC5335900 28252199

[phy271139-bib-0052] Rogers, G. W. , Brand, M. D. , Petrosyan, S. , Ashok, D. , Elorza, A. A. , Ferrick, D. A. , & Murphy, A. N. (2011). High throughput microplate respiratory measurements using minimal quantities of isolated mitochondria. PLoS One, 6, e21746. https://www.ncbi.nlm.nih.gov/pubmed/21799747 10.1371/journal.pone.0021746 PMC3143121 21799747

[phy271139-bib-0053] Rubio‐Aliaga, I. , & Krapf, R. (2022). Phosphate intake, hyperphosphatemia, and kidney function. Pflügers Archiv, 474, 935–947. 10.1007/s00424-022-02691-x PMC9338892 35511366

[phy271139-bib-0054] Sallis, R. E. (2009). Exercise is medicine and physicians need to prescribe it! British Journal of Sports Medicine, 43, 3–4. 10.1136/bjsm.2008.054825 18971243

[phy271139-bib-0055] Sawalla Guseh, J. , Lieberman, D. , & Baggish, A. (2022). The evidence for exercise in medicine–a new review series. NEJM Evidence, 1, EVIDra2100002. 10.1056/EVIDra2100002 38319204

[phy271139-bib-0056] Serrano, J. , Kondo, S. , Link, G. M. , Brown, I. S. , Pratley, R. E. , Baskin, K. K. , Goodpaster, B. H. , Coen, P. M. , & Kyriazis, G. A. (2025). A partial loss‐of‐function variant (Ile191Val) of the TAS1R2 glucose receptor is associated with enhanced responses to exercise training in older adults with obesity: A translational study. Metabolism, 162, 156045. https://www.ncbi.nlm.nih.gov/pubmed/39393515 10.1016/j.metabol.2024.156045 PMC11637915 39393515

[phy271139-bib-0057] Shi, T. H. , Wang, B. , & Natarajan, S. (2020). The influence of metabolic syndrome in predicting mortality risk among US adults: Importance of metabolic syndrome even in adults with Normal weight. Preventing Chronic Disease, 17, E36. 10.5888/pcd17.200020 PMC7279064 32441641

[phy271139-bib-0058] Smith, J. A. B. , Murach, K. A. , Dyar, K. A. , & Zierath, J. R. (2023). Exercise metabolism and adaptation in skeletal muscle. Nature Reviews. Molecular Cell Biology, 24, 607–632. 10.1038/s41580-023-00606-x PMC10527431 37225892

[phy271139-bib-0059] Supplements NIoHOoD Phosphorus Fact Sheet for Health Professionals. https://ods.od.nih.gov/factsheets/Phosphorus‐HealthProfessional/

[phy271139-bib-0060] Sylow, L. , Tokarz, V. L. , Richter, E. A. , & Klip, A. (2021). The many actions of insulin in skeletal muscle, the paramount tissue determining glycemia. Cell Metabolism, 33, 758–780. 10.1016/j.cmet.2021.03.020 33826918

[phy271139-bib-0061] Tani, Y. , Sato, T. , Yamanaka‐Okumura, H. , Yamamoto, H. , Arai, H. , Sawada, N. , Genjida, K. , Taketani, Y. , & Takeda, E. (2007). Effects of prolonged high phosphorus diet on phosphorus and calcium balance in rats. Journal of Clinical Biochemistry and Nutrition, 40, 221–228. 10.3164/jcbn.40.221 PMC2275768 18398500

[phy271139-bib-0062] Ugrica, M. , Bettoni, C. , Bourgeois, S. , Daryadel, A. , Pastor‐Arroyo, E. M. , Gehring, N. , Hernando, N. , Wagner, C. A. , & Rubio‐Aliaga, I. (2021). A chronic high phosphate intake in mice is detrimental for bone health without major renal alterations. Nephrology, Dialysis, Transplantation, 36, 1183–1191. 10.1093/ndt/gfab015 33515264

[phy271139-bib-0063] U.S. Department of Agriculture ARS . (2024). Nutrient intakes from food and beverages: Mean amounts consumed per individual, by male/female and age, what we eat in America, NHANES August 2021‐2023. USDA. https://www.ars.usda.gov/northeast‐area/beltsville‐md‐bhnrc/beltsville‐human‐nutrition‐research‐center/food‐surveys‐research‐group/docs/wweianhanes‐overview/

[phy271139-bib-0064] Vorland, C. J. , Stremke, E. R. , Moorthi, R. N. , & Hill Gallant, K. M. (2017). Effects of excessive dietary phosphorus intake on bone health. Current Osteoporosis Reports, 15, 473–482. 10.1007/s11914-017-0398-4 PMC5693714 28840444

[phy271139-bib-0065] Wolfson, J. A. , Tucker, A. C. , Leung, C. W. , Rebholz, C. M. , Garcia‐Larsen, V. , & Martinez‐Steele, E. (2025). Trends in adults' intake of un‐processed/minimally processed, and ultra‐processed foods at home and away from home in the United States from 2003‐2018. The Journal of Nutrition, 155, 280–292. 10.1016/j.tjnut.2024.10.048 PMC11795699 39489418

[phy271139-bib-0066] Wu, H. , & Ballantyne, C. M. (2017). Skeletal muscle inflammation and insulin resistance in obesity. The Journal of Clinical Investigation, 127, 43–54. 10.1172/JCI88880 PMC5199705 28045398

[phy271139-bib-0067] Xie, Z. , Bailey, A. , Kuleshov, M. V. , Clarke, D. J. B. , Evangelista, J. E. , Jenkins, S. L. , Lachmann, A. , Wojciechowicz, M. L. , Kropiwnicki, E. , Jagodnik, K. M. , Jeon, M. , & Ma'ayan, A. (2021). Gene set knowledge discovery with Enrichr. Current Protocols, 1, e90. 10.1002/cpz1.90 PMC8152575 33780170

[phy271139-bib-0068] Zhang, H. , Zhou, X. D. , Shapiro, M. D. , Lip, G. Y. H. , Tilg, H. , Valenti, L. , Somers, V. K. , Byrne, C. D. , Targher, G. , Yang, W. , Viveiros, O. , Opio, C. K. , Mantzoros, C. S. , Ryan, J. D. , Kok, K. Y. Y. , Jumaev, N. A. , Perera, N. , Robertson, A. G. , Abu‐Abeid, A. , … Zheng, M. H. (2024). Global burden of metabolic diseases, 1990–2021. Metabolism, 160, 155999. 10.1016/j.metabol.2024.155999 39151887

[phy271139-bib-0069] Zhou, W. , Barkow, J. C. , & Freed, C. R. (2017). Running wheel exercise reduces alpha‐synuclein aggregation and improves motor and cognitive function in a transgenic mouse model of Parkinson's disease. PLoS One, 12, e0190160. 10.1371/journal.pone.0190160 PMC5741244 29272304

[phy271139-bib-0070] Zhu, J. Y. , & Guo, L. (2025). Exercise‐regulated lipolysis: Its role and mechanism in health and diseases. Journal of Advanced Research, 75, 291–309. 10.1016/j.jare.2024.11.031 PMC12536597 39613256

[phy271139-bib-0071] Zoll, J. , Sanchez, H. , N'Guessan, B. , Ribera, F. , Lampert, E. , Bigard, X. , Serrurier, B. , Fortin, D. , Geny, B. , Veksler, V. , Ventura‐Clapier, R. , & Mettauer, B. (2002). Physical activity changes the regulation of mitochondrial respiration in human skeletal muscle. The Journal of Physiology, 543, 191–200. 10.1113/jphysiol.2002.019661 PMC2290497 12181291

